# Literary Notes

**Published:** 1899-01

**Authors:** 


### LITERARY NOTES.

THE Palisade Manufacturing Co., of Yonkers, N. Y., has issued a beautiful antique brochure, entitled Chirurgy and Medicine—Ambrose Paré, 1579. It is a brief synopsis, illustrated with the quaint figures, of the original of "the works of that famous chirurgeon Ambrose Parey out Latine and compared with the French, by Tho Johnson, London. Printed by Richard Cates and Willi Dugard, and are to be sold by John Clarke, entering into Mercer Chappell, 1649." It is selected from one of the original volumes now in the congressional library at Washington, which now stained and worn by the lapse of over 300 years. These stains are immolated, as far as possible in the printing of this extract from the quaint book. It can be had on application to the publishers.

*Climate* is the title of a new medical magazine, the first number of which is dated November, 1898. It announces that it is devoted to the relation of climate, mineral springs, diet, preventive medicine, race, occupation, life insurance and sanitary science to disease—all for one dollar a year. The initial number certainly presents an excellent appearance. It is edited by Dr. S. Claiborne Martin, Jr., and a list of ten associates or collaborators and is published at 3408 Franklin Avenue, St. Louis, Mo.

THE *Metaphysical Magazine*, "devoted to the advanced thought of the age—scientific, philosophic, psychic and occult—Leander Edward Whipple, editor," is about the most bigoted specimen of magazine literature extant, if the September issue for 1898 can be considered a fair specimen of its struggle with the few simple problems that it has allotted to itself, according to the above extract from its title page. Its attack upon the state board of medical examiners of Massachusetts is about as silly as it is idle, and we should be sorry for the board if such a senseless editorial column was not directed against it. To be defended by it would be at least suspicious.

MR. E. N. DICKERSON, of the New York bar, recently made an elaborate argument before the commission to revise the patent and trade-mark laws of the United States, a printed copy of which has been sent us by the Farbenfabriken of Elberfeld Co., New York. It is in the main a contention for the protection of medicinal drugs and preparations manufactured abroad, and the argument is ingeniously and logically presented. There is, of course, a vast difference between the secret patented formulæ of the ready-made medical mixtures of the nostrum vender, and the legitimate chemical products of a scientific laboratory that have proved beneficial to the sick. Physicians of training, of course, discriminate between the two, but it will do them no harm to read Mr. Dickerson's presentation of the subject. The brochure can be obtained by addressing the above named house, P. O. Box, 2660.

---

THE *Medical Dial* is the name of a new medical periodical lately established at Minneapolis, the first number of which appeared in December and presents an excellent appearance. Dr. J. W. Macdonald is the editor and his associate is Dr. C. K. Bartlett.

---

THE Practice of Obstetrics, by American Authors, edited by Professor Charles Jewett, M. D., is forthcoming at an opportune time. Obstetrics progresses so rapidly, particularly in this country, that a completely new work by acknowledged masters of all the subjects it comprises will be welcomed. It will be a practical book as its title indicates, yet its suitability for the obstetrician will not lessen its value as a text-book. Indeed it numbers among its contributors the professors in many of the leading colleges, so that it wlll doubtless have widespread success in the student world. The publishers, Lea Brothers & Co., have spared nothing in typography and illustration compatible with issuing the volume at a price within the reach of all.

---

MESSRS. M. J. BREITENBACH COMPANY, 100 Warren street, New York, are sending out to the medical profession their year-book for 1899. This is a most useful daily memorandum for the desk and none who has heretofore used it could afford to do without it. If any physician has been overlooked he can obtain the book by addressing the publishers.

